# Perioperative Challenges in a Septic Shock Patient With Extensive Splanchnic and Extra-Splanchnic Vein Thrombosis Up to the Right Atrium

**DOI:** 10.7759/cureus.32935

**Published:** 2022-12-25

**Authors:** Chun Lei Tan, Louis Ng

**Affiliations:** 1 Department of Anaesthesia & Surgical Intensive Care, Changi General Hospital, Singapore, SGP

**Keywords:** unfractionated heparin, ivc thrombus, anti-factor xa, pseudo-heparin resistance, splanchnic venous thrombosis

## Abstract

Venous thromboembolism (VTE) has a significant disease burden worldwide and comprises deep vein thrombosis (DVT) and pulmonary embolism (PE). DVT most commonly occurs in the lower extremities, very rarely it can present in the splanchnic venous circulation or inferior vena cava or both.

We report an unusual presentation of a 68-year-old woman with septic shock secondary to ischemic bowel, complicated by non-tumor related, extensive right hepatic vein thrombosis extending up to the inferior vena cava (IVC) and right atrium. She underwent bowel resection surgery emergently and was started on systemic anticoagulation in the perioperative period after an extensive evaluation and resuscitation. She was managed by a multidisciplinary team during her admission and was discharged after four weeks.

This case poses an interesting therapeutic challenge to the team as there is little literature to guide treatment in a critically ill patient with ischemic bowel, septic shock with extensive splanchnic and IVC thrombosis who also had likely pseudo-heparin resistance with artifactual activated partial thromboplastin time (aPTT) values. This case report seeks to share our experience of a multidisciplinary and patient-centric approach in this rare presentation of a disease spectrum.

## Introduction

Venous thromboembolism (VTE) has a significant disease burden worldwide [1] and the most common presentations are deep vein thrombosis (DVT) in the lower extremities [2] and pulmonary embolism (PE). Mortality and morbidity associated with VTE is widely known [3,4]. Splanchnic vein thrombosis (SVT) is a rare presentation of VTE [5]. To our knowledge, this is the first documented case report of such extensive thrombosis in both the splanchnic venous circulation and inferior vena cava in a patient with poly-microbial bacteremia that is non-tumor related that had been treated and discharged. We present the following case report in accordance with the CARE reporting checklist [6].

## Case presentation

A 68-year-old Indian woman with a past medical history of type 2 diabetes mellitus, inflammatory bowel disease (IBD) on budesonide and anemia presented to our emergency department with a four-day history of abdominal pain, fever and diarrhea. She travelled from India to Singapore one week ago to visit his son. The team had limited information regarding her history of IBD and anaemia, and was told by the daughter-in-law that she had been managing herself well in India by herself, and denied any hospital admission or surgery. At the emergency department, her vitals were: blood pressure 75/50 mmHg, heart rate 112 beats/min, respiratory rate 22 breaths/min, temperature 39.4°C and peripheral oxygen saturation of 73% on room air. On physical examination, her abdomen was soft and distended with generalized tenderness. A nasogastric tube was inserted and 900ml of gastric fluid was aspirated upon insertion. She was started on peripheral noradrenaline infusion and converted to central noradrenaline infusion after central venous line insertion after appropriate fluid resuscitation. She did not exhibit any history of arterial or venous thromboembolism, malignancy or clonal hematological disorder.

**Figure 1:**
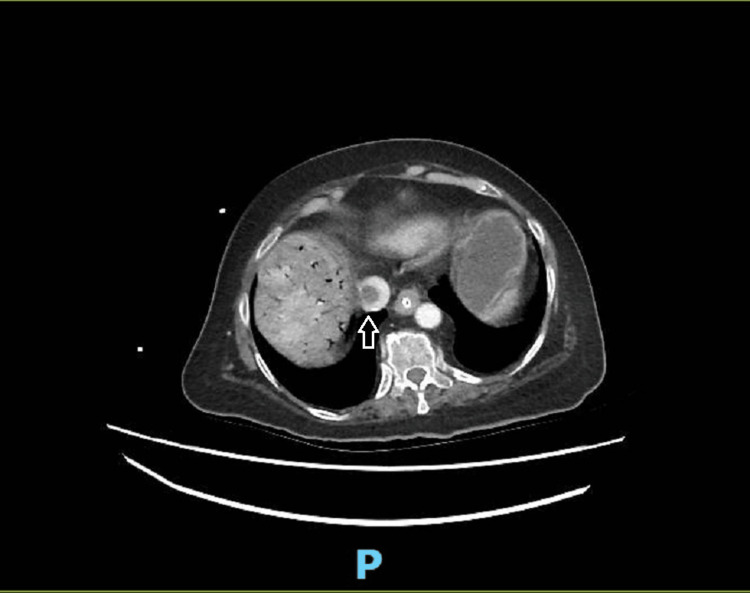
Contrast-enhanced CT (sagittal view) in the portal venous phase on day of admission showing thrombus in the inferior vena cava (IVC).

**Figure 2:**
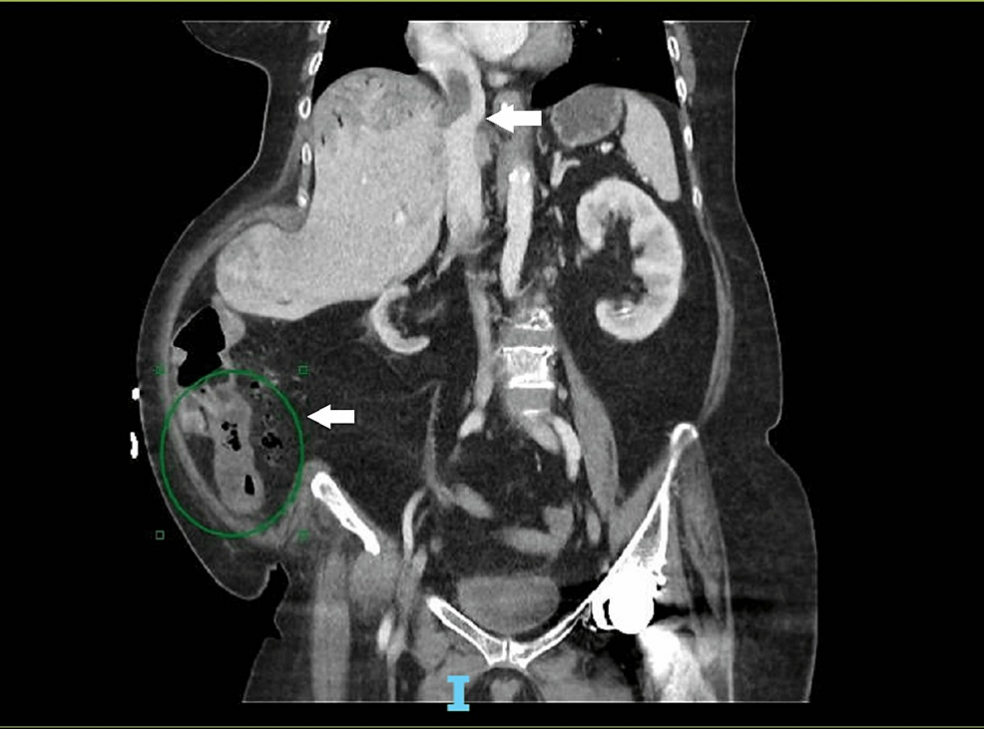
Contrast-enhanced CT (coronal view) in the portal venous phase done on the day of admission showing thrombus in the right hepatic vein extending into the inferior vena cava (IVC) and downstream terminal ileum is collapsed and associated mural oedema.

Laboratory tests were significant for elevated inflammatory markers with deranged renal panel and elevated lactate. We also detected elevated alkaline phosphatase and gamma-glutamyl transferase on the liver function test. Values are summarized in Table 1. An urgent contrast-enhanced computed tomography (CT) was performed to assess the cause of septic shock. It showed extensive portal venous gas with patchy attenuation of the liver with right hepatic vein thrombosis extending up to the inferior vena cava and possibly right atrium. There were dilated small bowel loops with transition point in the distal ileum, associated with upstream small bowel and an obstructing foreign body. Gas was noted in the mesenteric veins, with normal enhancement of the main portal veins and mesenteric arteries. Images are shown below in Figure 1 and Figure 2. The patient and family members were counselled for immediate surgery in the emergency operating theater (OT). Despite her elevated risk for peri-operatively in view of her unstable hemodynamics and clinical findings, both patient and her family members agreed to proceed with the life-saving surgery.

**Table 1:** Laboratory data on admission * SI unit conversion: to convert PO2 to kPa, multiply by 0.133.

Variable	Result	Reference range
Urea, serum (mmol/L)	4.9	2.8 - 7.7
Sodium, serum (mmol/L)	133	135 - 145
Potassium, serum (mmol/L)	4.4	3.5 - 5.3
Chloride, serum (mmol/L)	96	96 - 108
Bicarbonate, serum (mmol/L)	15	19 - 31
Glucose, serum (mmol/L)	8.9	3.1 - 7.8
Creatinine, serum (mmol/L)	133	50 - 90
Albumin, serum (g/L)	37	37 - 51
Bilirubin total, serum (mmol/L)	9.5	5.0 - 30.0
Alkaline phosphatase, serum (U/L)	181	32 - 103
Alanine transaminase, serum (U/L)	51	10 - 55
Aspartate transaminase, serum (U/L)	77	10 - 45
Gamma-Glutamyl Transferase, serum (U/L)	232	5 - 50
Lactate, plain blood (mmol/L)	11.17	0.5 - 2.2
International normalized ratio	1.19	2.0 - 3.50
Activated partial thromboplastin time (s)	31.5	24.0 - 34.0
Prothrombin time (s)	12.6	9.5 - 11.5
Haemoglobin (g/dL)	14.4	11.5 - 15.0
Leucocyte count (x10^3 ^mL)	31.4	4.0 - 10.0
Platelet Count (x10^3 ^mL)	215	150 - 450

**Figure 3:**
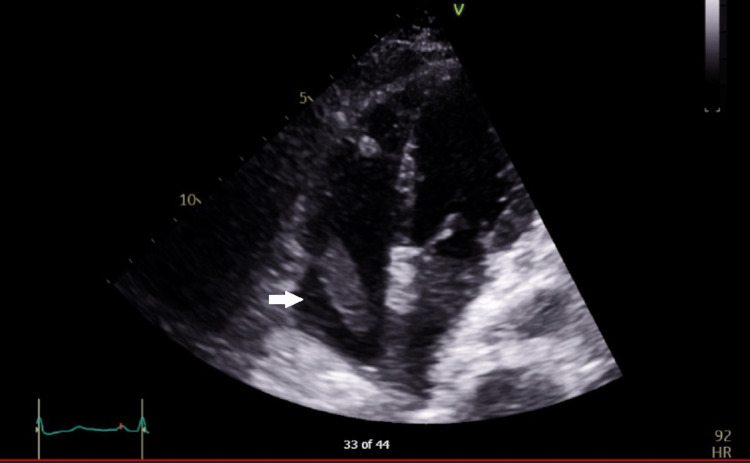
Transthoracic echocardiogram showed right atrium thrombus.

**Figure 4:**
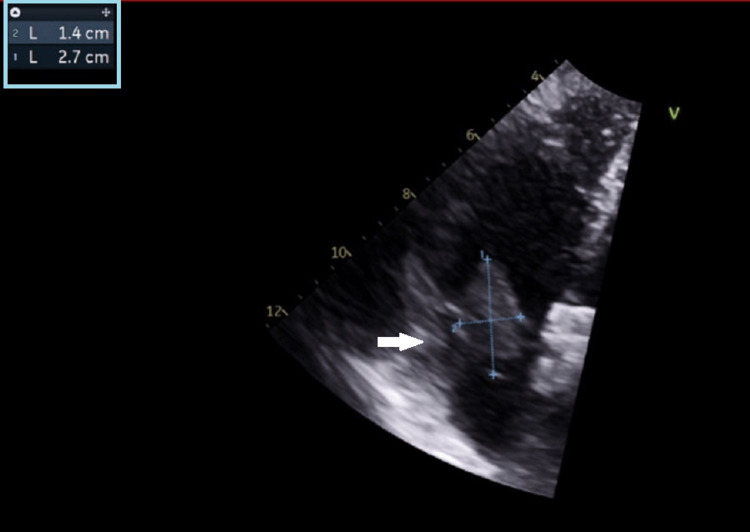
Transthoracic echocardiogram showed the size of the right atrium thrombus.

She was sent to the operating theatre from the emergency department and underwent exploratory laparotomy, right hemicolectomy and temporary abdominal closure. Cardio-stable induction was performed with fentanyl, lignocaine and ketamine and she was intubated after paralysis with rocuronium. Intraoperatively, she needed inotropic support of up to noradrenaline 0.6 mcg/kg/min and vasopressin 0.04 unit/min. She was given a total of 2 liters of crystalloids with no blood products. Findings upon entering the abdomen include clear ascites, no pus seen, terminal ileum noted to have patchy hemorrhagic infarcts suggestive of venous thrombosis, no evidence of ischemia/infarct suggestive of arterial thrombosis, and no obvious foreign body palpable in the distal ileum as mentioned in the CT scan. The surgeon performed resection of the ileum, followed by caecum and ascending colon as the caecum was noted to be dusky after initial distal ileum resection. The abdomen was temporarily closed in view of her high inotropic requirement intraoperatively and she was transferred intubated to the intensive care unit. In the ICU, she was monitored closely in terms of her fluid status and resuscitated while further investigations were done to evaluate the thrombosis. We managed to wean her inotropes down over the first 24 hours with goal-directed fluid therapy guided by FloTrac^TM^ system (Edwards Lifesciences, Irvine, CA, USA). Transthoracic echocardiography (TTE) was performed to assess her cardiac function and extent of the thrombus. It showed a preserved ejection fraction with a 2.7x1.4cm thrombus in the right atrium (RA). Images are shown in Figure 3 and Figure 4.

Her stay in the hospital had multi-disciplinary involvement including the primary colorectal surgeon, vascular surgeon, cardiothoracic surgeon and hematologist in the management of her extensive thrombus. An interventional radiologist was also consulted for the possibility of catheter-directed clot removal of the RA thrombus but was deemed not feasible due to the high risk of clot dislodgement. Surgical thrombectomy of RA thrombus was also explored by the cardiothoracic surgeon; however, this option carried high mortality and morbidity. After weighing the risks and benefits of various options versus that of systemic anticoagulation in view of the recent major abdominal surgery, she was prescribed a loading dose of heparin and started on an infusion of IV unfractionated heparin, titrated to a target-activated partial thromboplastin time (aPTT) of 50-70 seconds. The coagulation profile was checked every 4 hours as per our institution's protocol. For the first 24 hours, we had difficulty achieving therapeutic aPTT (as shown in Table 2) despite increasing the infusion rate as per protocol. We considered the possibility of heparin resistance and viscoelastic haemostatic assays (VHA) using ROTEM-Sigma^TM^ (Werfen Corporate, Barcelona, Spain) was performed to check for heparin effects. ROTEM^TM^ showed a significant prolongation of clotting time on INTEM and with reduction of clotting time on HEPTEM, signifying the presence of heparin effect. Activated clotting time (ACT) and anti-factor Xa (anti-Xa) heparin assay were used subsequently to determine the effects of heparin but there was discordance between ACT and anti-Xa result with ACT showing supra-therapeutic value with a sub-therapeutic Anti-Xa value (as shown in Table 2). Haematologist was consulted and it was decided that Anti-Xa is the only reliable test in view of the artefactual values of both ACT and aPTT values which were the more traditional methods in guiding heparin infusion for anti-coagulation. However, we faced the challenges that although Anti-Xa level was the preferred test, the test had a very long turnaround time at our institution. We eventually decided on checking the anti-Xa level once a day while running the heparin infusion at a lower infusion rate along with close monitoring of the patient clinically for bleeding or thrombosis.

**Table 2:** Selected laboratory data over the course of intensive care unit stay. *Patient on IV unfractionated heparin infusion.

Variable	Admission	POD1	POD2	POD3	Reference range
Urea, serum (mmol/L)	4.9	5.3	6.5	7.9	2.8 - 7.7
Sodium, serum (mmol/L)	133	132	140	139	135 - 145
Potassium, serum (mmol/L)	4.4	4.1	3.3	3.4	3.5 - 5.3
Chloride, serum (mmol/L)	96	98	100	102	96 - 108
Bicarbonate, serum (mmol/L)	15	19	25	24	19 - 31
Glucose, serum (mmol/L)	8.9	20.3	6.6	9.9	3.1 - 7.8
Creatinine, serum (mmol/L)	133	85	89	76	50 - 90
Albumin, serum (g/L)	37	20	33	32	37 - 51
Bilirubin total, serum (mmol/L)	9.5	7.9	28.6	26.7	5.0 - 30.0
Alkaline phosphatase, serum (U/L)	181	106	88	70	32 - 103
Alanine transaminase, serum (U/L)	51	310	207	149	10 - 55
Aspartate transaminase, serum (U/L)	77	755	223	109	10 - 45
Gamma-Glutamyl Transferase, serum (U/L)	232	126	77	52	5 - 50
Lactate, plain blood (mmol/L)	11.17	5.98	2.07		0.5 - 2.2
Procalcitonin (ng/ml)		>100			
International normalized ratio	1.19				2.0 - 3.50
Activated partial thromboplastin time (s)	31.5	50.7	30.5	>100	24.0 - 34.0
Prothrombin time (s)	12.6				9.5 - 11.5
Activated clotting time (s)			>1001	>1001	70 - 120
Anti-Xa Heparin Assay (units/ml)			0.11	0.12	0.6 - 1.0
Haemoglobin (g/dL)	14.4	10.2	9.3	9.3	11.5 - 15.0
Leucocyte count (x10^3 ^mL)	31.4	28.5	19.9	15.2	4.0 - 10.0
Platelet Count (x10^3 ^mL)	215	146	61	35	150 - 450

During her ICU stay, her platelet count started exhibiting a downward trend (as shown in Table 2); our impression was most likely sepsis-induced thrombocytopenia, thus heparin was continued throughout while we were waiting for the result of anti-hep PF4. Heparin-induced thrombocytopenia was ruled out when Anti hep PF4 came back negative. On postoperative day (POD) four, heparin infusion was stopped and she underwent a relook laparotomy, stoma creation uneventfully. She stayed anti-coagulation free till two days later as the surgeon was worried about post-operatively bleeding in view of thrombocytopenia. She was started on subcutaneous enoxaparin sodium (clexane) on day 3 after her definitive surgery. She stayed in ICU for a total of six days before discharging to a high-dependency unit with no major organ failures. The blood culture on admission was positive for extended-spectrum beta-lactamase (EBSL) producing Escherichia coli, Aeromonas, Klebsiella pneumoniae and Clostridium perfringens. She was treated with two weeks course of antibiotics (meropenem and ciprofloxacin) as per our antibiotic stewardship guidance program. Blood sampling was sent off for anti-cardiolipin IgG antibody, anti-nuclear antibody (ANA) and anti-double stranded DNA and was noted to be negative. The histology for her intra-operative specimens did not show any evidence of vasculitis, granuloma, dyspepsia or malignancy. Bilateral deep vein thrombosis screen for the lower limbs was also negative. No tumors were detected on her scans. Clexane was switched to rivaroxaban upon discharge from the hospital. Ideally, we would like to further evaluate her with the inheritable thrombophilia panels which include activated protein C (APC)/Factor V Leiden (FVL), prothrombin gene mutation, protein C and S, and anti-thrombin with repeat scans to evaluate the size of the thrombus but she had travelled back to India shortly after discharge from our hospital. A memo was given to her so that she can have her investigations done in India instead. The patient’s laboratory results are summarised in Table 2.

## Discussion

Venous thromboembolism (VTE) is the leading cause of mortality and morbidity affecting over millions of people globally. Annual incidence rates range from 0.75 to 2.69 per 1000 individuals in population in countries such as Western Europe, North America, Australia and Southern Latin America [7], and studies conducted in Asia have consistently reported lower rates of VTE than western population [8]. Significant chronic sequelae may occur in up to 50% of the patients including chronic thromboembolic pulmonary hypertension, post-thrombotic syndrome and post-PE syndrome with functional limitations. Splanchnic vein thrombosis (SVT) encompasses portal vein thrombosis, mesenteric vein thrombosis, splenic vein thrombosis and Budd-Chiari syndrome (BCS) [5]. Extrahepatic thrombosis such as inferior vena cava (IVC) thrombosis can present in the setting of isolated thrombus or propagation from the splanchnic circulation. The incidence of IVC thrombosis in patients with confirmed deep vein thrombosis (DVT) is 4-15% [9,10] and it is frequently associated with neoplastic disease. Our patient presented with spontaneous IVC and right atrium (RA) thrombus with a negative DVT scan and no evidence of primary or secondary malignancy.

Traditionally thrombus formation is associated with Virchow’s triad which consists of abnormalities in blood composition, vessel wall components, and blood flow [11]. This forms the basis for the aetiology and risk factors predisposing to the formation of SVT and IVC thrombosis. It can be classified into idiopathic/unprovoked and secondary/provoked based on the presence of causative triggers. We hypothesized that the presence of inflammatory bowel disease (IBD) with sepsis predisposes to venous thromboses in our patient owing to associated risks of hypovolemia, elevated inflammatory response and alteration in the balance of coagulation pathway. But the histopathology report of the intra-operative specimens proved otherwise. There was no evidence of IBD flare. There is considerable evidence to suggest the association of acquired infective aetiology with a variant of primary IVC thrombosis that is endemic in Nepal [12]. The pathogenesis of this thrombus formation is suspected to be a combination of thrombophlebitis and transient protein-S deficiency from bacteremia.

The primary objectives of treatment are targeted at reducing the risk of thrombus propagation, improving vessel recanalization and preventing complications such as pulmonary embolism, chronic portal hypertension and further local ischemia. Treatment options include medical, endovascular and surgical. Medical therapy with systemic anticoagulation is strongly recommended in patients without absolute contraindications [13,14]. Studies have shown that early commencement of anti-coagulation therapy promotes better vessel recanalization and aids in the prevention of re-thrombosis [15]. Our patient presented with relative contraindication as she had recent surgery with temporary closure of the abdomen, putting her at higher risk of post-operative bleeding. We explored both the endovascular and surgical options but they were deemed too high risk. After a careful risk-to-benefit evaluation, she was started on unfractionated heparin (UFH) infusion instead of low molecular weight heparin (LMWH) or vitamin K antagonist (warfarin), because of its shorter half-life, independence from renal function and reversibility by protamine sulphate in consideration for the urgent and unpredictable need to return to OT.

When UFH is used, the monitoring is usually by activated partial thromboplastin time (aPTT) due to the ease of automation, accessibility and cheaper cost. According to institution protocol, blood sampling is done after 4 hours since treatment onset and additional samplings at 4 hours interval for heparin dose adjustment. The target therapeutic range for UFH is 1.5-2 times the control aPTT. UFH is a highly negatively charged molecule and can bind to positively charged plasma proteins, proteins released from platelet and endothelial cell proteins and surfaces [16,17]. This explains the marked variability in its anticoagulant response among individual patients, which can be further exaggerated in the critically ill. The drawbacks we experienced in our case were confounding results and delay in reaching the therapeutic range despite titrating the infusion rate.

Heparin resistance was suspected in our case in view of the persistence of non-elevated aPTT values, but this was disproved by viscoelastic haemostatic assays (VHA) using ROTEM-Sigma^TM ^which demonstrated significant heparin effects. Critically-ill patients can develop hemostatic abnormalities, ranging from isolated thrombocytopenia, factors deficiencies to disseminated intravascular coagulopathy (DIC) [18]. This coagulation derangement can result in true or pseudo-heparin resistance. Heparin resistance is defined as the need for greater than 35000 units of heparin in 24 hours to reach therapeutic aPTT levels. True heparin resistance is observed when there is an inadequate response to the heparin in vivo. Our case might present as a case of pseudo-heparin resistance due to elevated factor VIII and fibrinogen levels with low to normal level of aPTT despite UFH infusion. Ideally, we would like to measure anti-thrombin III and factor VIII level but both tests are not available in our hospital.

To differentiate between true and pseudo-heparin assay, anti-factor Xa heparin assay is used instead [19]. Anti-Xa heparin assay is an alternative for UFH infusion monitoring due to its lack of susceptibility to interference from the elevated concentration of factor VIII or fibrinogen that result from acute phase reactions. However, despite its potential advantages over aPTT, it is more expensive and not readily available in all laboratories for testing at any time locally. Our blood samples were transported to an external laboratory and results were only available at least 6 hours later. In view of the challenges in getting results and lack of protocols with titration of therapy according to factor Xa levels, we performed blood samplings for both anti-factor Xa (anti-Xa) heparin assay and activated clotting time (ACT) for this patient in order to determine which test can be more easily referenced for therapeutic guidance. ACT could be performed in a hospital within a very short period of time as point-of-care testing while Anti-Xa levels will take up to 6 hours to return a result as it has to be shipped to an external laboratory. We found that there was no correlation between the ACT value, aPTT and anti-Xa. ACT is the most frequently used tool in extracorporeal therapies and percutaneous interventions to monitor the anticoagulation effect of high-dose UFH but evidence of its use to monitor low-dose UFH in ICU patients is sparse [20]. To date, studies have not been able to show the correlation of ACT with anti-Xa for low-dose UFH in ICU patients, as this group of patients usually exhibit coagulation derangements with abnormal parameters such as platelet, fibrinogen and hematocrit levels that could influence the performance of the ACT system. Eventually, we decided to continue the UFH infusion for up to 4 hours at a lower rate despite the sub-therapeutic anti-Xa level due to the limitation in obtaining the result post titration prior to her definitive surgery and restarted LMWH thereafter.

## Conclusions

We presented a case of septic shock patient with ischemic bowel complicated by right hepatic vein thrombosis up to inferior vena cava (IVC) and right atrium that was treated with systemic anticoagulation. Splanchnic vein thrombosis (SVT) and IVC thrombosis are rare manifestations of venous thromboembolism (VTE) that have significant mortality and morbidity. This was further complicated by artifactual readings of activated partial thromboplastin time (aPTT) and activated clotting time (ACT) values which were typically used to monitor the effect of heparin anticoagulation. Our case highlighted the disadvantages of aPTT-guided heparin therapy, the usefulness of viscoelastic haemostatic assays (VHA) using ROTEM-Sigma^TM^ in confirming the heparin effect, the need for a robust Anti-Xa protocol in managing anticoagulation in patients who are critically ill and the importance of a multidisciplinary patient-centric approach when tackling challenging clinical cases with resource and knowledge constraints so that early diagnosis, prompt and appropriate treatments can be performed to promote the best outcome for patients.
